# Implementing public involvement standards in cerebral palsy register research

**DOI:** 10.3389/fresc.2022.903167

**Published:** 2022-11-17

**Authors:** Claire Kerr, Karen McConnell, Helen Savage, Monica Acheson

**Affiliations:** ^1^ School of Nursing & Midwifery, Queen's University Belfast, Medical Biology Centre, Belfast, United Kingdom; ^2^ School of Health Sciences, Ulster University, Newtownabbey, United Kingdom; ^3^ Public Contributor to Northern Ireland Cerebral Palsy Register, School of Nursing & Midwifery, Queen's University Belfast, Medical Biology Centre, Belfast, United Kingdom

**Keywords:** public involvement, cerebral palsy, register, standards, experience

## Abstract

**Background:**

In 2018, the National Institute for Health Research launched Draft Standards for Public Involvement in Research. The Northern Ireland Cerebral Palsy Register (NICPR) was competitively selected as a “test-bed” project to pilot the Draft Standards over a one-year period.

**Aim:**

This perspective paper aims to describe the NICPR's experience of piloting the Draft Standards for Public Involvement in Research, highlighting successes and challenges.

**Method:**

Three of the six Draft Standards were piloted from April 2018 to April 2019: Standard 2 “working together”, Standard 4 “communications” and Standard 5, “impact”.

**Results:**

Implementation of Standard 2 resulted in formation of a dedicated Public Involvement Group. Standard 4 was implemented by revision of the NICPR's Privacy Notice and development of the NICPR website. Standard 5 was not implemented during the test-bed pilot period.

**Discussion:**

Benefits of use of the Draft Standards in cerebral palsy register research included development of relationships, improving quality, accessibility and relevance of NICPR materials, increasing skills and confidence, networking opportunities, advocating for others and feeling empowered to shape cerebral palsy research. Challenges included administrative issues, absence of dedicated and sustained funding, limitations in the availability and applicability of public involvement training and the time required for meaningful public involvement.

**Conclusions:**

Standards for Public Involvement provide a useful framework for structuring and embedding meaningful public involvement. Sustained, authentic public involvement in cerebral palsy register research ensures that people affected by the condition are empowered to engage, inform, develop and lead research that meets their needs.

## Introduction

Patient and public involvement in research (PI) is when research is “carried out “with” or “by” members of the public rather than “to”, “about” or “for” them”(1). It denotes an active partnership between patients, carers and members of the public with researchers. PI is an expectation in many fields of research but perhaps most notably in disability research where participation in decision-making has been long advocated from a democratic perspective. PI can help empower people who use health and social care services by providing an opportunity to influence the commissioning, design, conduct and dissemination of research. PI can improve research relevance and quality by ensuring that the views of people with lived experience are represented and that outcomes of importance to them are addressed.

In the UK, the National Institute for Health Research (NIHR) funds, enables and delivers health and social care research in partnership with the National Health Service, universities, patients, the public and other stakeholders (2). Although PI was always core to the work of the NIHR (3), renewed focus in this area was evident from 2013 onwards (3–5), with Draft Standards for Public Involvement in Research being published in 2017. The Draft Standards were piloted by 40 different organisations and people from May 2018–May 2019, including 10 “test-bed” projects (6). The “test-bed” projects were purposively selected following an open call for expressions of interest. Test-beds ranged from University or Hospital Trust Research Departments, to networks within Royal Colleges, to established patient-researcher collaborations in specific health conditions. Piloting involved test-bed projects implementing their chosen Draft Standards and providing frequent feedback and opinions on the implementation to the NIHR throughout the one-year period. Feedback from the pilots and test-beds was incorporated into the finalised Standards for Public Involvement, launched in November 2019 (7). The Standards aim to improve the quality and consistency of public involvement in research, effectively providing a description of what “good” public involvement looks like. Six Standards are defined (see Table 1). NIHR suggest that the Standards can be used in multiple ways, for example, as a framework for researchers to plan or review PI, to encourage reflection and learning, or for public and community groups to consider their involvement in PI.

**Table 1:** NIHR standards for public involvement (7).

Standard	Description
Inclusive Opportunities	Offer public involvement opportunities that are accessible and that reach people and groups according to research needs.
Working Together	Work together in a way that values all contributions, and that builds and sustains mutually respectful and productive relationships.
Support and Learning	Offer and promote support and learning opportunities that build confidence and skills for public involvement in research.
Governance	Involve the public in research management, regulation, leadership and decision making.
Communications	Use plain language for well-timed and relevant communications, as part of involvement plans and activities.
Impact	Seek improvement by identifying and sharing the difference that public involvement makes to research.

The Northern Ireland Cerebral Palsy Register (NICPR) (8) was one of the ten “test-bed” projects selected to pilot the Draft Standards. The NICPR is a confidential record of children with cerebral palsy in Northern Ireland. It provides a systematic approach to monitoring and surveillance of the condition in the region and supports research nationally (9–12) and internationally (13, 14). Cerebral palsy encompasses “a group of permanent disorders of the development of movement and posture” that cause limitation in activities and are due to a non-progressive brain injury *in utero* or very early in life (15). Cerebral palsy is a life-long condition. Prevalence of cerebral palsy in adults is estimated to be 2.38/1,000, similar to that of multiple sclerosis or Parkinson's disease (10). A recent data linkage study between the NICPR and routine hospital system data demonstrated higher rates of hospital admissions and outpatient appointments for children and young people with cerebral palsy compared to the general population (11). It is thus important that people with cerebral palsy, and their families, friends and carers are included in health and social care research to ensure it is relevant and meets the needs of the population. The NICPR has had parent and young person representation on its Advisory Committee from its inception in the early 1990's, however, in more recent years we have sought to integrate PI across all the activities of the register. Our selection as a “test-bed” (6) to pilot the Draft Standards for Public Involvement provided an excellent opportunity to reflect on our PI and examine how we could grow and sustain this into the future to ensure that the activities of the NICPR were useful to people with the condition.

This paper summarises the experiences of the NICPR team in piloting the Draft Standards for Public Involvement as part of the “test-bed” project. We report what, why and how we implemented the Draft Standards and then reflect on the challenges and benefits of the process.

## Piloting the draft standards for public involvement – what, why and how?

**Figure 1:**
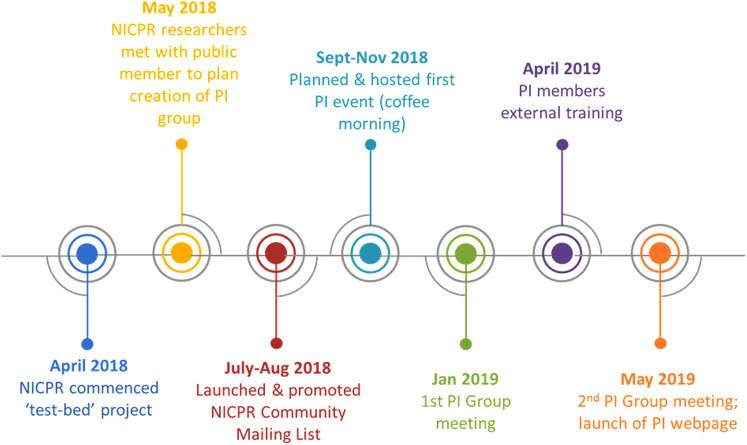
Timeline of public involvement group creation.

We aimed to embed three of the six Draft Standards for Public Involvement over a 12-month period. Standard 2, “Working Together”, was selected because a key objective of the NICPR at the time was to ensure public involvement was integrated into all of the register's activities. Standard 2 was implemented by establishing a group of people with cerebral palsy, their families, friends or carers, to a dedicated PI group that would advise on all PI activities and guide the direction of NICPR research. Creation of the PI group was facilitated by setting up a community mailing list for people interested in NICPR news, events, research and ways to get involved in our activities (http://eepurl.com/c–F7T). A flyer (https://bit.ly/2Gpk4Ik) was developed and used to promote the mailing list *via* the NICPR website, social media platforms and various stakeholders, including voluntary sector organisations and healthcare professionals. Subscribers to the community mailing list were invited to attend a community coffee morning to meet NICPR researchers and find out more about becoming involved with the work of the NICPR. This resulted in the creation of the PI group comprising two researchers, two adults with cerebral palsy and one parent of a young child with the condition. At the PI group's inaugural meeting the group developed their terms of reference and identified immediate training needs (chairing meetings, understanding the role of PI in research). Consequently, training was sourced and attended by the majority of the group. During the second meeting the PI group finalised their terms of reference and defined key objectives for the following year. Figure 1 summarises the processes and timeline in creating the PI group.

Standard 4, “Communications”, aligned with the NICPR objective to update and develop NICPR resources using a variety of communication methods for various stakeholder groups. The update and development of NICPR resources was overseen by the PI group to ensure materials were “jargon-free”. The PI group successfully revised the NICPR's Privacy Notice to reduce the amount of legal jargon (https://bit.ly/2PXrbh3) and the Family Information Leaflet (https://bit.ly/3nc3hO4). The group also agreed content for a new “Get Involved” section on the NICPR's website (https://bit.ly/2GtKetc), and developed new resources including a Child Information Leaflet (https://bit.ly/3fbKuOF), animated information video (https://youtu.be/1WvonsURK8M) and animated video about becoming involved in NICPR activities (https://youtu.be/Uv-LgzifUPc).

Finally, as part of the test-bed pilot of the draft Standards for Public Involvement, the NICPR also aimed to embed Standard 5, “Impact”, by developing standardised evaluation processes to establish the impact of PI on NICPR activities and to demonstrate to public members that their voices are heard. Evaluating impact also aligned with the increased focus on measuring the impact of research beyond academia (16). Unfortunately, PI group development of standardised evaluation processes was not completed during the test-bed pilot period as discussed below.

## Discussion

### Challenges associated with implementing the NIHR draft standards for public involvement

One of the main challenges experienced as part of the NICPR's test-bed experience was the perception that the Draft Standards were more complicated than necessary. This made PI appear “overly academic” to the members of our PI group. The adults with cerebral palsy in our PI group were keen to share their personal stories and to hear the stories of others, however they felt that the language used in the Draft Standards might deter others from getting involved, which they viewed as converse to the whole ethos of PI whereby everyone's story is of importance, and no-one should be precluded from being involved. Our PI group believed strongly that PI should be uncomplicated and accessible to maximise inclusion and diversity. Fortunately, following the test-bed pilot phase, some of the language of the Draft Standards was amended, resulting in the current Standards for Public Involvement (7) being clearer. Our PI group welcomed these changes as they felt that the Standards were now more accessible for non-academic audiences, easier to understand and operationalise, and consequently meaningful PI was perceived to be attainable.

A further challenge identified by the NICPR was the limited funding available for PI early in the research cycle, a challenge also identified in the literature (17). Whilst funding for PI can be incorporated into research grant applications, it can be difficult to obtain dedicated funding for early PI activities that are required to support such applications. Yet, early, pre-protocol funding is required to embed PI in the research process (18). In addition, funding for PI needs to be sustained to ensure quality and consistency of PI activities and to enable people from different backgrounds, abilities and experiences to feel motivated and valued in their PI activities (19). Since the publication of the current Standards for Public Involvement (7), many academic institutions and large funding bodies (e.g., UKRI, Wellcome) offer seed funding to facilitate early PI activities, but these opportunities are not available in all institutions, are typically of short duration, and are often not accessible to all researchers, such as those on short-term contracts.

As a small research team, the NICPR identified adequate time and staff resource as additional challenges to implementing the Draft Standards. For example, the process of creating the NICPR's PI group took longer than anticipated, due in part to organisational requirements aligned with the introduction of General Data Protection Regulations (GDPR) in 2018. Developing meaningful PI is time-intensive and it can be difficult to allocate adequate resources, particularly in smaller research teams that have very real, competing academic demands such as teaching, the need to publish and maintain research funding income. However, the perspective of our PI group is that allocating adequate time and resources for PI activities is vital to ensure meaningful involvement, maintain and increase our research quality and relevance and, as also identified by others, to avoid unintentional unethical consequences, such as tokenism and poor communication (20).

Finally, the greatest challenge faced by our PI group was in implementation of Standard 5, “impact”, evidenced by the fact that we did not achieve this during the test-bed time-frame. The need to measure the “impact” of our PI, and what and why we might measure, particularly at this very early stage of formalising our PI processes, was discussed frequently by the group. The adults with lived experience of cerebral palsy felt that measurement of involvement and impact might “tokenise” PI and deter other contributors with lived experience. They perceived that simple, easily measured indicators (such as number of PI meetings and contributors, diversity of contributors) would diminish the richness and authenticity of their contributions and inhibit dialogue and co-learning between those with lived experience and researchers. This perspective echoes with a rights-based approach that frames “PI” as an end in itself (21), as opposed to PI as a means of facilitating “better research”. Our group continue to explore ways of capturing the impact of PI that are valued by all members of our PI group, drawing on the numerous systematic reviews (22, 23), tools (24), frameworks (25) and conceptualisations (26) available in the literature.

### Perceived benefits associated with implementing the NIHR draft standards for public involvement

Using the Draft Standards for Public Involvement provided a flexible framework for the NICPR to structure its PI activities and embed meaningful PI in cerebral palsy register research. The framework offered a structured means of listening to people's lived experiences and ensuring that experiences were listened to authentically. The structured approach reassured PI group members with lived experience, facilitated a safe space to share experiences and knowledge, and ensured all contributions were valued. The flexibility offered by the Draft Standards permitted the NICPR to prioritise implementation of the Draft Standards most relevant to our work at that point in time, guiding our PI activities in alignment with our objectives and organisational requirements. Effectively, we used the Draft Standards as a benchmark, allowing us to reflect upon and monitor progress in our PI and affording an opportunity for continuous improvement.

In practical terms, implementing the NIHR Draft Standards for Public Involvement enhanced clarity and accessibility of NICPR resources and communications. New videos and resources developed whilst implementing Standard 4, “Communications”, were well received by the local cerebral palsy community, highlighting the importance of providing information in a variety of methods, suitable for different audiences. Therefore, the Standards can be used to ensure provision of clear, jargon-free communication which is widely recognised as key to successful PI (18, 20).

The members of the NICPR PI group with lived experience of cerebral palsy reported personal benefits because of their involvement with the PI group and the test-bed project. Reported benefits included increased confidence from sharing personal experiences, networking and public speaking. PI group members with lived experience of cerebral palsy felt empowered in sharing their stories and advocating for others with the condition. Whilst sharing stories in this way can inspire research by generating new ideas (27), it can also encourage other service users to come forward to find out more about getting involved as a contributor. For example, from our experience, one public contributor sharing their story at an informal family coffee morning, inspired several others to become involved in NICPR research and activities.

## Conclusion and recommendations

Our experience as a test-bed piloting the Draft Standards for Public Involvement in Research provided a timely opportunity to evaluate our approach to PI. We aspired to embed and sustain PI across the work of our cerebral palsy register and the Draft Standards provided a flexible framework to reflect on our work to date and structure short and medium terms plans for PI. Standards 2 and 4, “working together” and “communications”, were successfully implemented, whereas Standard 5, “impact”, was not achieved within the test-bed pilot due mainly to time constraints, but also in relation to a developing understanding of why, how and what we might consider in the context of PI impact.

Our PI group members with lived experience of cerebral palsy valued the structure that the Draft Standards provided. They believed that the Draft Standards could be used as lens to frame and assess the mission, vision and values of an organisation, a research team, or an individual research project. Further, they felt that the Draft Standards provided a structure to improve transparency in both the planning and operationalisation of research that they believed would lead to improved research quality and relevance and increase engagement from other PI contributors. One PI group member commented “Standards do not limit PI, instead, they allow it to grow” but also cautioned that “PI is not static” and that the “Standards provide a framework for constant reflection and evaluation for improvement.” It is these sentiments that we take forward in the PI activities of the NICPR. We summarised our “test-bed” journey in a lay report available on the NICPR website (https://bit.ly/3ws16va).

PI is increasingly embedded in health and disability research and service provision in the UK and internationally. Literature related to PI has grown exponentially in recent years and it can feel overwhelming to keep abreast of best practice in the area. The UK Standards for Public Involvement (7) provide a rigorously developed, flexible framework for good PI in research that can be used by members of the public, researchers, organisations that deliver or support research, and research funders. We suggest that the Standards, and their accompanying exemplar “Implementation Stories” (28), are an excellent starting point for anyone interested in public involvement in research.

## Data Availability

The original contributions presented in the study are included in the article/Supplementary Material, further inquiries can be directed to the corresponding author/s.
